# Mitochondrial tRNA-Derived Diseases

**DOI:** 10.3390/ijms262412023

**Published:** 2025-12-13

**Authors:** Antonia Petropoulou, Nikolaos Kypraios, Dimitra Rizopoulou, Adamantia Kouvela, Alexandros Maniatis, Katerina Anastasopoulou, Alexandra Anastogianni, Theodoros Korfiatis, Katerina Grafanaki, Vassiliki Stamatopoulou, Constantinos Stathopoulos

**Affiliations:** 1 Department of Biochemistry, School of Medicine, University of Patras, 26504 Patras, Greece; 2 Department of Dermatology, School of Medicine, University of Patras, 26504 Patras, Greece

**Keywords:** mitochondrial tRNA, mtDNA mutations, mt-tRNA modifications, human diseases

## Abstract

Mitochondrial tRNA genes are critical hotspots for pathogenic mutations and several mitochondrial diseases. They account for approximately 70–75% of disease-causing mtDNA variants despite comprising only 5–10% of the mitochondrial genome. These mutations interfere with mitochondrial translation and affect oxidative phosphorylation, resulting in remarkably heterogeneous multisystem disorders. Under this light, we systematically reviewed PubMed, Scopus, and MITOMAP databases through October 2025, indexing all clinically relevant pathogenic mt-tRNA mutations classified by affected organ systems and underlying molecular mechanisms. Approximately 500 distinct pathogenic variants were identified across all 22 mt-tRNA genes. Beyond typical syndromes like MELAS, MERRF, Leigh syndrome, and Kearns–Sayre syndrome that are linked to mt-tRNA mutations, they increasingly implicate cardiovascular diseases (cardiomyopathy, hypertension), neuromuscular disorders (myopathies, encephalopathies), sensory impairment (hearing loss, optic neuropathy), metabolic dysfunction (diabetes, polycystic ovary syndrome), renal disease, neuropsychiatric conditions, and cancer. Beyond sequence mutations, defects in post-transcriptional modification systems emerge as critical disease mechanisms affecting mt-tRNA function and stability. The mutations on tRNA genes described herein represent potential targets for emerging genome editing therapies, although several translational challenges remain. However, targeted correction of pathogenic mt-tRNA mutations holds transformative potential for precision intervention on mitochondrial diseases.

## 1. Introduction

Although the fundamental role of tRNA in translation of the genetic messages has been recognized since the 1950s, the connection between tRNA mutations and human disease emerged rather recently, when MELAS (Mitochondrial Encephalomyopathy, Lactic Acidosis, and Stroke-like episodes) was linked to mutations in the mitochondrial tRNA^Leu^ gene (*MTTL1*) [1]. Shortly thereafter, mutations in tRNA^Lys^ were identified as the underlying cause of MERRF (Myoclonic Epilepsy with Ragged Red Fibers) [2], establishing the paradigm that defects in mitochondrial tRNAs could result in severe human pathologies. Since these initial discoveries, mutations in multiple tRNA genes have been implicated in various diseases, with considerable phenotypic heterogeneity observed even when the same tRNA gene is affected. The clinical outcome and severity of such mutations remain notoriously difficult to predict [3,4,5].

Mitochondrial DNA (mtDNA) is a compact circular molecule encoding 37 genes: 13 polypeptides of the electron transport chain, 22 tRNAs, and 2 rRNAs [6]. These mt-tRNAs adopt a conserved cloverleaf secondary structure and are indispensable for mitochondrial protein synthesis [7,8]. Despite representing only about 5% of the mitochondrial genome, mt-tRNA genes harbor nearly 70–75% of all reported pathogenic mtDNA mutations [9], underscoring their critical importance in mitochondrial translation and oxidative phosphorylation (OXPHOS). According to the MITOMAP database, a substantial number of the identified pathogenic point mutations in mtDNA occur within tRNA genes, with over 500 pathogenic variants having been reported to date (accessed on 27 November 2025). Because mt-tRNA genes are highly conserved, due to their essential structural roles, even single-nucleotide substitutions can destabilize secondary structure and disrupt canonical base pairing. Mutations within these regions frequently destabilize tRNA structure, disrupt canonical base pairing, and compromise secondary structure integrity [10]. As tRNAs are irreplaceable mediators of amino acid delivery during translation [8], pathogenic mutations typically impair mtDNA translation, leading to global deficiencies in respiratory chain complex activity.

While many nucleotide variations in mtDNA represent benign polymorphisms, pathogenic mutations manifest in a remarkably broad spectrum of mitochondrial disorders with multi-organ involvement. Among these, the A3243G mutation in tRNA^Leu^, encoding tRNA^Leu(UUR)^, stands out as the most frequently encountered pathogenic mtDNA variant. This mutation exhibits extraordinary phenotypic variability, affecting the central and peripheral nervous systems, skeletal muscle, heart, endocrine organs, gastrointestinal tract, and skin. Clinical presentations range from well-defined syndromes to isolated symptoms or overlapping syndromic features, complicating diagnostic approaches. Recent studies have further highlighted cardiac complications and risks during pregnancy and the perinatal period associated with this variant [11]. Despite decades of research, the molecular mechanisms underlying many mt-tRNA mutations remain incompletely understood, and effective therapeutic interventions are still missing [12,13].

Despite the exponential growth of genomic data and the increasing recognition of mitochondrial perturbations in severe clinical phenotypes, a significant gap remains in the systematic documentation and clinical characterization of pathogenic mutations in mitochondrial tRNA genes. Although numerous studies have identified mt-tRNA mutations, no comprehensive classification integrating genotype–phenotype and mechanistic data has been published recently. While individual case reports and disease-specific studies continue to emerge at a rapid pace, comprehensive efforts to collate, categorize, and contextualize the full spectrum of mt-tRNA-associated pathologies have been notably limited. This fragmentation of knowledge hinders both diagnostic precision and our understanding of genotype-phenotype correlations in mitochondrial medicine. Herein, we present a comprehensive and systematically updated review that consolidates all clinically relevant pathogenic mutations in mitochondrial tRNA genes reported to date. By organizing these mutations according to their associated clinical phenotypes—from typical mitochondrial syndromes to rare and emerging disease entities—we provide a critical resource that underscores the contribution of mt-tRNA dysfunction to human pathology. This classification not only facilitates a deeper mechanistic understanding of disease pathogenesis but also serves as a practical framework for clinical diagnosis, genetic counseling, and the identification of novel therapeutic targets in mitochondrial disorders.

## 2. Search Strategy

This review was conducted using a systematic approach to identify relevant literature on mitochondrial tRNA mutations and associated therapeutic strategies. A comprehensive search was performed using PubMed (https://pubmed.ncbi.nlm.nih.gov/, accessed on 30 October 2025) and Scopus (https://www.scopus.com, accessed on 30 October 2025) databases, covering all publications up until October 2025. We systematically integrated all reported pathogenic mt-tRNA mutations based on data from MITOMAP (www.mitomap.org), and PubMed databases up to 2025, classifying them according to affected organ systems and underlying molecular mechanisms. This classification framework aims to establish mechanistic links between mt-tRNA structural defects and disease phenotypes, thereby facilitating diagnostic interpretation and informing therapeutic design strategies. Literature searches used the terms: “mitochondrial tRNA”, “mt-tRNA mutations”, “pathogenic mt-tRNA variants” combined with gene-specific nomenclature for all 22 mt-tRNA genes and disease terms like “MELAS”, “MERRF”, etc. Studies were included if they reported mt-tRNA mutations with clinical phenotypes, provided genetic/biochemical/functional evidence of pathogenicity, were peer-reviewed English publications, and were documented in MITOMAP. Exclusion criteria included common polymorphisms without disease association, insufficient clinical data, or lack of pathogenicity evidence. Mutations were classified as pathogenic based on genetic evidence, biochemical defects, functional validation, evolutionary conservation, structural disruption of critical tRNA domains, and MITOMAP classification.

## 3. Mitochondrial Genome

**Figure 1:**
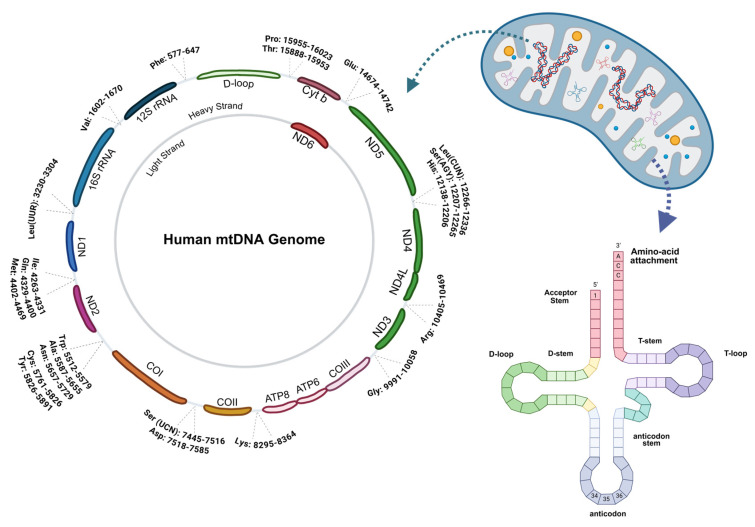
Human mitochondrial DNA genome with genes and tRNA structure. (Created in BioRender. https://BioRender.com/c3wwytf (accessed on 12 November 2025)).

Mitochondria are essential organelles present in nearly all human cells, with the notable exception of erythrocytes. These powerhouses of cellular energy are responsible for adenosine triphosphate (ATP) generation through oxidative phosphorylation. Each cell typically harbors several hundred to thousands of copies of the mitochondrial genome—a circular molecule spanning 16,569 base pairs in humans [14]. This compact genome encodes 37 genes critical for cellular function. Thirteen polypeptides are dedicated to the electron transport chain: seven subunits (ND1, ND2, ND3, ND4, ND4L, ND5, and ND6) comprise complex I, Cyt b contributes to complex III, three proteins (Cytochrome c oxidase (COX1, COX2, and COX3)) form complex IV, and ATP6 and ATP8 constitute part of complex V. Beyond these protein-coding genes, the mitochondrial genome also encodes 22 tRNAs and 2 rRNAs, a 12S and a 16S, all indispensable for mitochondrial protein synthesis (Figure 1) [14]. Among the 22 mt-tRNA genes, leucine and serine are each represented by two distinct tRNAs, and these mt-tRNAs adopt a conserved cloverleaf secondary structure comprising an acceptor arm, D-arm, anticodon stem, variable region, and TψC loop, with an average length of approximately 73 nucleotides [7,8,15,16]. Within this translational machinery, mt-tRNAs work in concert with aminoacyl-tRNA synthetases, elongation factors, and ribosomes to ensure proper gene expression [14]. Mitochondrial translation fidelity can be compromised through diverse mechanisms. Single nucleotide substitutions within codons may impair the accuracy, efficiency, or stability of protein synthesis. Structural defects in mt-tRNAs caused by mutations can result in molecular instability, increased susceptibility to degradation, and impaired aminoacylation—a process that critically depends on precise recognition by aminoacyl-tRNA synthetases. The vulnerability of mitochondrial DNA to mutation is striking. According to MITOMAP, mt-tRNA mutations represent the most frequent category among mitochondrial variants. Remarkably, every tRNA gene has been reported to harbor at least one mutation, many detectable using next-generation sequencing technologies [14]. The high mutational burden observed in mitochondrial DNA results from multiple mechanisms: replication-associated errors as well as direct damage from reactive oxygen species and other DNA-damaging agents. Notably, inherited mtDNA variants have been implicated in the pathogenesis of systemic lupus erythematosus (SLE) and multiple sclerosis, while mitochondrial ROS production is markedly elevated in SLE and across several cancer types [17,18,19].

Mitochondrial diseases (MDs) constitute a clinically and genetically heterogeneous group of disorders characterized by oxidative phosphorylation defects. The distribution and heteroplasmic load of mtDNA mutations profoundly influence disease severity and progression rates across different cell types [20]. Tissues with high energy demand, including the central nervous system, skeletal muscle, cardiac muscle, retina, and cochlea, are disproportionately affected given that they are rich in mitochondria. Despite constituting only 5–10% of the mitochondrial genome, mt-tRNA genes represent critical hotspots for pathogenic point mutations. The underlying mechanisms include impaired folding, reduced structural stability, inefficient aminoacylation, and disrupted post-transcriptional modifications [21]. These modifications, particularly at the wobble position of the anticodon, are essential for accurate genetic decoding and tRNA stability [22].

## 4. Human Diseases Associated with mt-tRNA Mutations

### 4.1. Typical Mitochondrial Syndromes

**Figure 2:**
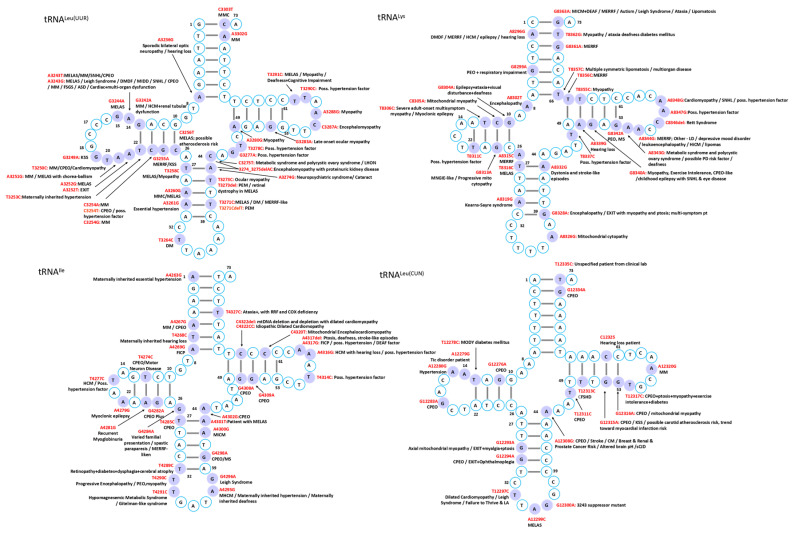
Representation of human mitochondrial tRNA^Leu(UUR)^, tRNA^Lys^, tRNA^Ile^ and tRNA^Leu(CUN)^ in their characteristic cloverleaf secondary structures. Pathogenic mutations are highlighted in light purple. Reference tRNA sequences were obtained from the tRNA database (https://gtrnadb.org/index.html (accessed on 30 November 2025)), while information on pathogenic variants was sourced from MITOMAP (www.mitomap.org). Reported disease associations for each mutation are also indicated.

**Figure 3:**
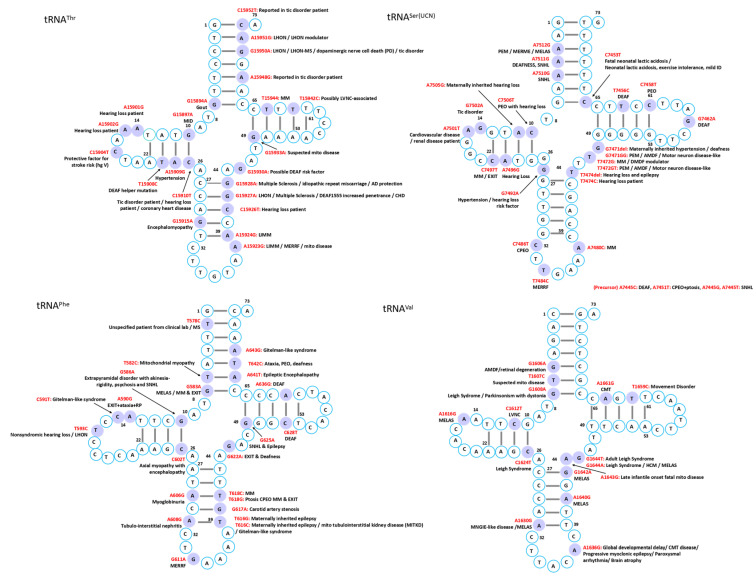
Representation of human mitochondrial tRNA^Thr^, tRNA^Ser(UCN)^, tRNA^Phe^ and tRNA^Val^ in their characteristic cloverleaf secondary structures. Pathogenic mutations are highlighted in light purple. Reference tRNA sequences were obtained from the tRNA database (https://gtrnadb.org/index.html) (accessed on 12 November 2025), while information on pathogenic variants was sourced from MITOMAP (www.mitomap.org). Reported disease associations for each mutation are also indicated.

**Figure 4:**
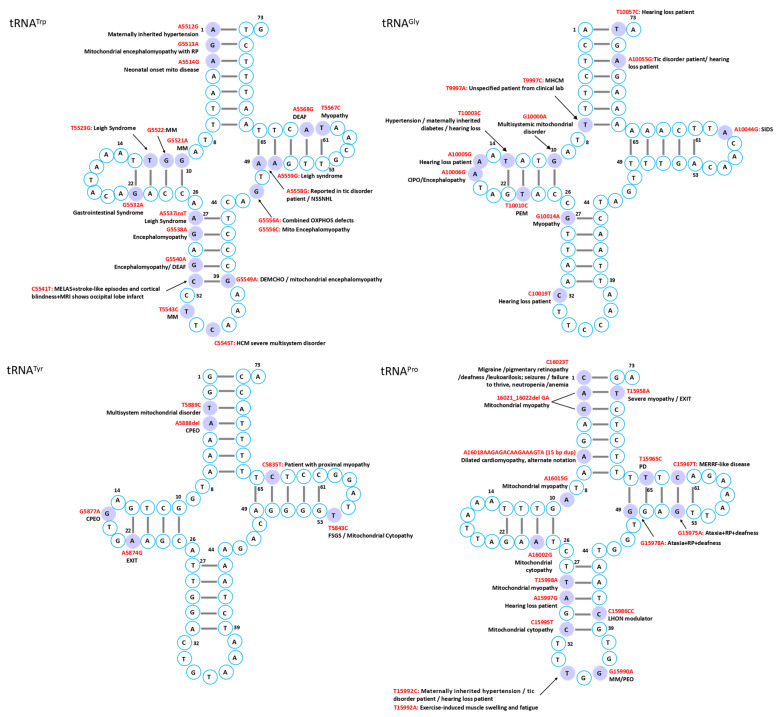
Representation of human mitochondrial tRNA^Trp^, tRNA^Gly^, tRNA^Tyr^ and tRNA^Pro^ in their characteristic cloverleaf secondary structures. Pathogenic mutations are highlighted in light purple. Reference tRNA sequences were obtained from the tRNA database (https://gtrnadb.org/index.html), while information on pathogenic variants was sourced from MITOMAP (www.mitomap.org). Reported disease associations for each mutation are also indicated.

**Figure 5:**
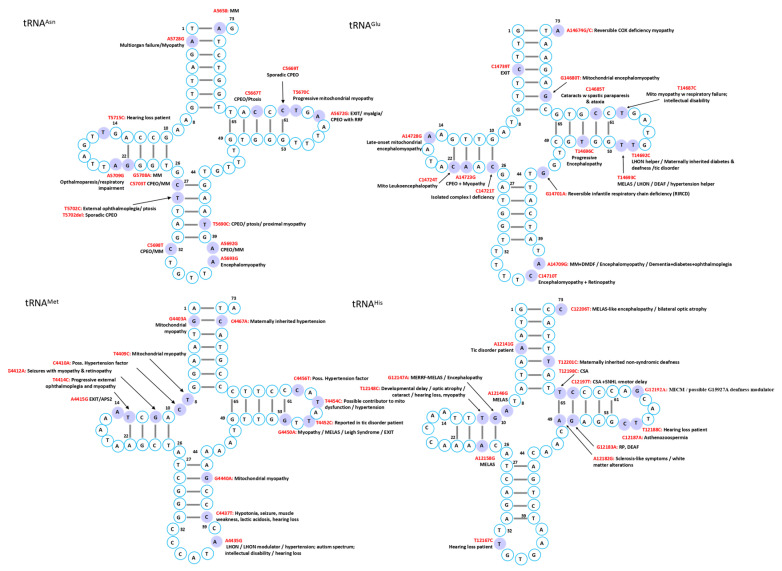
Representation of human mitochondrial tRNA^Asn^, tRNA^Glu^, tRNA^Met^ and tRNA^His^ in their characteristic cloverleaf secondary structures. Pathogenic mutations are highlighted in light purple. Reference tRNA sequences were obtained from the tRNA database (https://gtrnadb.org/index.html), while information on pathogenic variants was sourced from MITOMAP (www.mitomap.org). Reported disease associations for each mutation are also indicated.

**Figure 6:**
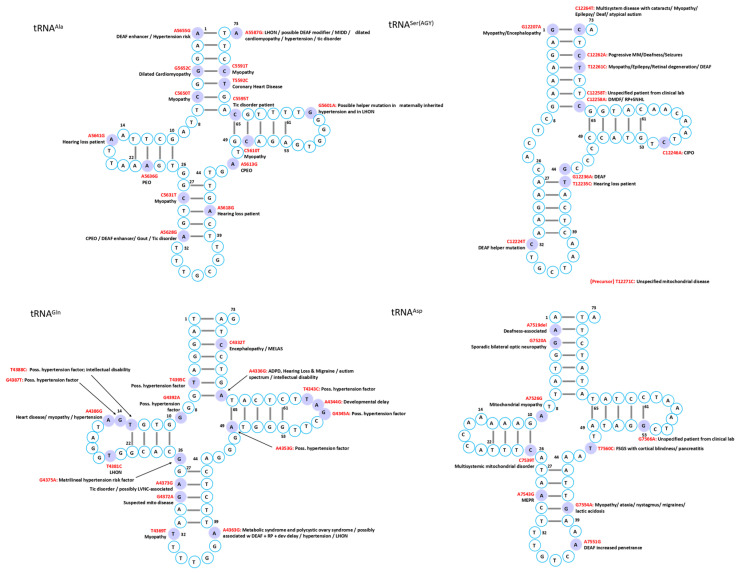
Representation of human mitochondrial tRNA^Ala^, tRNA^Ser(AGY)^, tRNA^Gln^ and tRNA^Asp^ in their characteristic cloverleaf secondary structures. Pathogenic mutations are highlighted in light purple. Reference tRNA sequences were obtained from the tRNA database (https://gtrnadb.org/index.html), while information on pathogenic variants was sourced from MITOMAP (www.mitomap.org). Reported disease associations for each mutation are also indicated.

**Figure 7:**
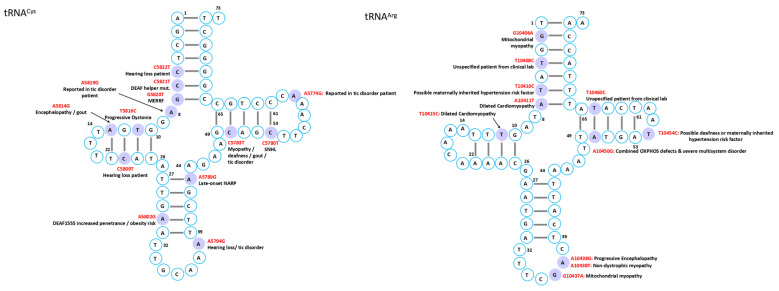
Representation of human mitochondrial tRNA^Cys^ and tRNA^Arg^ in their characteristic cloverleaf secondary structures. Pathogenic mutations are highlighted in light purple. Reference tRNA sequences were obtained from the tRNA database (https://gtrnadb.org/index.html. accessed on 12 November 2025), while information on pathogenic variants was sourced from MITOMAP (www.mitomap.org). Reported disease associations for each mutation are also indicated.

Mutations in mitochondrial tRNAs are linked to a broad spectrum of disorders characterized by diverse clinical manifestations and multi-organ involvement. While several syndromes have been associated with defined genetic mutations, the phenotypic heterogeneity often complicates diagnosis, though certain characteristic features can facilitate recognition in typical cases (Figure 2, Figure 3, Figure 4, Figure 5, Figure 6 and Figure 7).

#### 4.1.1. MELAS

MELAS is a maternally inherited disorder most frequently caused by the A3243G mutation in the tRNA^Leu^ gene, encoding tRNA^Leu(UUR)^ [23]. Despite its prevalence in approximately 1 in 400 individuals, clinical manifestations occur in only ~3.5 per 100,000, reflecting remarkably low penetrance [24,25]. A possible hypothesis is that this variant disrupts the stability of the native tRNA structure, potentially reducing aminoacylation efficiency and altering decoding fidelity. Although the precise molecular mechanism remains to be experimentally validated, disruption of conserved D-loop interactions could promote non-native conformations. Consistent with this model, structural studies have shown that pathogenic mutations in mt-tRNA^Leu(UUR)^ can induce the formation of aberrant dimeric species [26], supporting the broader concept that misfolded mt-tRNAs may contribute to mitochondrial dysfunction. These structural perturbations disrupt mitochondrial translation, impair oxidative phosphorylation, and ultimately cause cellular energy failure [27]. Clinically, MELAS typically presents in children or young adults with a constellation of symptoms, including headache, hemiparesis, cortical blindness, hemianopia, seizures, and lactic acidosis [28]. While A3243G accounts for most cases, additional mtDNA mutations—including other tRNA variants and complex I mutations—contribute to the considerable phenotypic variability observed in this syndrome [29,30]. The MITOMAP database documents numerous additional mutations implicated in MELAS pathogenesis, including A12146G, A12158G, and C12206T in tRNA^His^; A3252G, G3244A, T3258C, T3291C, C3256T, A3260G, and A3251G in tRNA^Leu^; A12299C in tRNA^Leu(CUN)^; T8316C in tRNA^Lys^; A4301T in tRNA^Ile^; G4450A in tRNA^Met^; G583A in tRNA^Phe^; A1616G, A1640G, G1642A, and A1630G in tRNA^Val^; and C5541T in tRNA^Trp^, underscoring the remarkable genetic heterogeneity underlying this syndrome.

#### 4.1.2. MERRF

In contrast to MELAS, MERRF is most associated with the A8344G mutation in tRNA^Lys^, which similarly impairs mitochondrial translation and results in energy deficiency in affected tissues [2]. The syndrome typically manifests in the second or third decade of life and is characterized by myoclonic epilepsy, cerebellar ataxia, muscle weakness, and the pathognomonic ragged-red fibers in skeletal muscle that reflect abnormal mitochondrial accumulation. Although the A8344G mutation in tRNA^Lys^ is the most frequent genetic cause, the mutational spectrum extends beyond this classic variant. Other reported mutations include A8296G, T8356C, G8361A, G8363A, G611A, and G3255A, some of which are associated with overlapping syndromes such as MELAS or Kearns–Sayre [31]. Additional MERRF-associated mutations in MITOMAP database encompass C5820A in tRNA^Cys^, A8315C in tRNA^Lys^, G4284A in tRNA^Ile^, G12147A in tRNA^His^, G15967A in tRNA^Pro^, and A15923G in tRNA^Thr^, further expanding the genetic heterogeneity of this disorder.

#### 4.1.3. Chronic Progressive External Ophthalmoplegia (CPEO) and Kearns–Sayre Syndrome (KSS)

CPEO and KSS represent two of the most common mitochondrial disorders affecting ocular and multisystem function. CPEO is characterized by progressive eyelid drooping and impaired eye movements, with approximately 60% of cases resulting from large-scale mtDNA deletions, while others arise from multiple deletions or point mutations in tRNA genes, particularly tRNA^Leu^ and tRNA^Leu(CUN)^ [32]. A diverse array of pathogenic variants has been associated with CPEO in MITOMAP, including T3250C and C3254T in tRNA^Leu^; T4274C, T4285C, G4309A, and G4298A in tRNA^Ile^; G12276A, G12294A, T12311C, and G12315A in tRNA^Leu CUN)^; C5703T, C5698T, and A5692G in tRNA^Asn^; A5628G in tRNA^Ala^; 5885delT and G5877A in tRNA^Tyr^; and G8342A in tRNA^Lys^. Recently, a novel T14677C mutation in tRNA^Glu^ has expanded the genetic spectrum of CPEO [33]. The genetic heterogeneity of CPEO is further illustrated in MITOMAP by additional pathogenic variants, including T5613C in tRNA^Ala^; A5690G, G5667A, A5702del, and G5669A in tRNA^Asn^; A4302G, G4308A, G4282A, and A4267G in tRNA^Ile^, with T4274C additionally linked to motor neuron disease and G4298A to multiple sclerosis; G12283A, G12334A, G12316A, and T12317C in tRNA^Leu(CUN)^; C15990T in tRNA^Pro^; G7486A in tRNA^Ser(UCN)^; C5877T and T5888del in tRNA^Tyr^; and T642C, T618C, and T618G in tRNA^Phe^. These mutations lead to defective mitochondrial translation and progressive external ophthalmoplegia.

KSS is a more severe mitochondrial disorder characterized by progressive external ophthalmoplegia, pigmentary retinopathy, and onset before the age of 20, often accompanied by cardiac conduction defects, cerebellar ataxia, and elevated cerebrospinal fluid protein [34]. Although large-scale deletions remain the primary genetic cause, tRNA point mutations such as G3249A and G3255A in tRNA^Leu^ have been reported in KSS or KSS-like phenotypes [34,35], highlighting that tRNA mutations represent an additional pathogenic mechanism worthy of diagnostic consideration. Other mutations associated with KSS in MITOMAP include A8319G in tRNA^Lys^ and G12315A in tRNA^Leu(CUN)^.

#### 4.1.4. Leigh Syndrome

Leigh syndrome is a progressive neurodegenerative disorder typically presenting in infancy or early childhood with developmental regression, hypotonia, respiratory abnormalities, and symmetrical basal ganglia and brainstem lesions [36]. Multiple pathogenic variants in mitochondrial tRNA genes have been implicated in MITOMAP, including the A3243G mutation in tRNA^Leu^ which impair mitochondrial protein synthesis. Notably, the G8363A mutation in tRNA^Lys^ was identified in blood and muscle from a 13-month-old girl with classical Leigh syndrome, whereas her mother, carrying the same mutation at lower heteroplasmy levels, developed late-onset MERRF, providing a compelling illustration of how mutation load influences phenotypic expression [37]. Additional genetic lesions include a single T insertion at nucleotide 5537 in tRNA^Trp^ in children with severe cytochrome-c oxidase deficiency, homoplasmic C1624T mutations in tRNA^Val^ in families with multiple neonatal deaths, a G1644A point mutation in tRNA^Val^ associated with adult-onset disease, and pathogenic variants in tRNA^Ile^ and tRNA^Leu(CUN)^ [36,38,39,40,41,42]. The genetic complexity of Leigh syndrome is further exemplified by additional pathogenic variants in MITOMAP, including T12297C in tRNA^Leu(CUN)^, G4450A in tRNA^Met^, A5559G, and T5523G in tRNA^Trp^, and G1644T, G1608A, C1624T, and G1644A in tRNA^Val^, demonstrating that Leigh syndrome can arise from mutations across diverse mt-tRNA genes.

#### 4.1.5. Mitochondrial Neurogastrointestinal Encephalopathy (MNGIE)

While MNGIE is classically caused by biallelic TYMP mutations leading to thymidine accumulation and mtDNA instability, mt-tRNA point mutations can produce clinically indistinguishable MNGIE-like phenotypes. These include gastrointestinal dysmotility, cachexia, ophthalmoplegia, peripheral neuropathy, and leukoencephalopathy. The A1630G mutation in tRNA^Val^, for instance, has been reported in patients with MNGIE-like features and demonstrates clear impairment of mitochondrial function in cybrid studies [38,43,44,45]. MNGIE-like phenotypes have also been associated with G8313A in tRNA^Lys^ [46].

### 4.2. Cardiovascular Diseases

The heart, being among the most mitochondria-rich organs [47], is particularly vulnerable to mt-tRNA mutations, which frequently disrupt tRNA folding, processing, or modification, ultimately compromising respiratory chain complex activity and exacerbating mitochondrial dysfunction (Figure 2, Figure 3, Figure 4, Figure 5, Figure 6 and Figure 7) [12].

#### 4.2.1. Cardiomyopathy

Cardiomyopathy encompasses a spectrum of structural and functional cardiac abnormalities, including hypertrophic (HCM), dilated (DCM), arrhythmogenic (AC), and left ventricular noncompaction (LVNC), with DCM being the most prevalent form [48,49]. Multiple mt-tRNA mutations have been implicated across these subtypes. The T3271C variant in tRNA^Leu(UUR)^ has been associated with hypertrophic cardiomyopathy in an Italian family [50], while pathogenic variants in tRNA^Ile^, including T4277C, A4295G, A4300G, C4320T, and A4317G, represent recognized hotspots in MITOMAP in fatal infantile cardiomyopathy. The A3243G mutation in tRNA^Leu(UUR)^, although classically linked to MELAS, is also frequently associated with hypertrophic cardiomyopathy and conduction defects [51]. Additional pathogenic variants associated with hypertrophic cardiomyopathy in MITOMAP include C5652G in tRNA^Ala^, A4316G in tRNA^Ile^, C5545T in tRNA^Trp^, A8348G in tRNA^Lys^, and G3242A in tRNA^Leu^, all of which impair mitochondrial translation and contribute to cardiac energetic failure. Similarly, the T3250C mutation shows a strong association with hypertrophic cardiomyopathy, underscoring the critical importance of cardiac surveillance in at-risk families and highlighting D-loop mutations in tRNA^Leu(UUR)^ as disease hotspots.

The mutational landscape extends to dilated cardiomyopathy, where several mt-tRNA variants have been identified. These include A10411T and T10415C in MT-TR, C4322CC and C4322del in tRNA^Ile^, T12297C in tRNA^Leu(CUN)^, and the 15 bp duplications T16018TTCTCTGTTCTTTCAT and T16032TTCTCTGTTCTTTCAT in tRNA^Pro^, demonstrating the critical role of tRNA integrity in maintaining cardiac contractile function [12,52,53,54,55,56]. In one family with maternally inherited DCM, a novel mt-tRNA^Ser(AGY)^ A12265G and a known mt-tRNA^Cys^ G5821A mutation impaired mitochondrial function, reduced ATP production, and increased reactive oxygen species (ROS) and Ca^2+^ levels [55]. Other mt-tRNA mutations, such as T8306C in tRNA^Lys^, which disrupts D-arm base pairing, have been associated with myocardial infarction and cardiomyopathy [12].

The A3243G mutation has also been reported in a patient with LVNC, complete heart block, nail-patella syndrome, and mitochondrial myopathy, and twelve mtSNPs have been identified in LVNC cohorts [57].

#### 4.2.2. Essential Hypertension

Essential hypertension (EH, MIM145500) is a major cardiovascular disorder and a leading risk factor for coronary heart disease, stroke, and kidney failure, contributing to approximately 7.6 million deaths annually [58,59]. Several mt-tRNA mutations have been linked to EH in Han Chinese families, including tRNA^Ile^ A4263G, mt-tRNA^Ser(UCN)^ 7471delC, tRNA^Met^/tRNA^Gln^ A4401G, and mt-tRNA^Ala^ C5601T [60,61,62,63]. These mutations reduce steady-state tRNA levels or aminoacylation efficiency, interfere with end processing and CCA addition, or disrupt chemical modifications, thereby impairing mitochondrial translation and contributing to disease pathogenesis [64]. The mt-tRNA^Gln^ T4386C mutation, identified in two Chinese pedigrees (one also carrying mt-tRNA^Ala^ C5601T), was associated with increased disease penetrance, while A14696G and A14693G mutations disrupt tRNA^Glu^ metabolism, potentially contributing to EH [65,66].

Notably, hypertension and dyslipidemia often co-occur, suggesting shared pathogenic mechanisms. In one family, maternally inherited hypertension, hypercholesterolemia, and hypomagnesemia were linked to a uridine-to-cytidine substitution 5′ to the tRNA^Ile^ anticodon, a conserved site critical for anticodon loop stability [67]. The MITOMAP database documents numerous additional mt-tRNA variants associated with essential hypertension risk in various populations, including T5655C and T5587C in tRNA^Ala^; T10410C in MT-TR; multiple tRNA^Gln^ variants (C4375T, A4343G, A4395G, C4345T, C4387A, C4392T, T4353C, A4388G); T4314C in tRNA^Ile^; A3261G, T3253C, G3277A, T3278C, and T3290C in tRNA^Leu^; A8347G, T8311C, and T8337C in tRNA^Lys^; C4467A, C4410A, C4456T, T4454C, and A4435G in tRNA^Met^; A5512G in tRNA^Trp^; and A15909G in tRNA^Thr^.

#### 4.2.3. Coronary Artery Disease and Cerebrovascular Disorders

Mitochondrial tRNA genes are frequent sites of pathogenic variants in coronary artery disease (CAD). The C15910T mutation in mt-tRNA^Thr^ co-segregates with maternally inherited coronary heart disease and disrupts tRNA processing, mitochondrial translation, and oxidative phosphorylation in cardiac cells. Additional mtDNA mutations linked to CAD include tRNA^Ile^ T4291C, tRNA^Leu^ T12285C and A12308G, tRNA^Thr^ G15927A, tRNA^Pro^ T15968C, and tRNA^Ala^ A5592G, which impair tRNA folding, aminoacylation, or protein synthesis, leading to reduced oxidative phosphorylation, increased ROS, and disturbed cardiac energetics [10,68,69,70,71,72].

Mt-tRNA mutations also contribute to cerebrovascular vulnerability by impairing protein synthesis and oxidative phosphorylation. The A12308G polymorphism in mt-tRNA^Leu(CUN)^ increases stroke risk among A3243AG carriers [73], while the pathogenic A1630G variant in mt-tRNA^Val^, reported in a patient with stroke, hearing loss, and short stature, disrupts mitochondrial respiration and respiratory chain complex function [44]. Additional mt-tRNA mutations in MITOMAP linked to cardiovascular and renal disease include T7501A in tRNA^Ser(UCN)^ and G617A in tRNA^Phe^ (associated with carotid artery stenosis), further expanding the genetic landscape of mitochondrial contributions to atherosclerotic disease.

Collectively, these mt-tRNA mutations impair tRNA stability, aminoacylation, post-transcriptional modification, or folding, disrupting mitochondrial translation, compromising respiratory chain function, increasing ROS, altering Ca^2+^ homeostasis, and ultimately driving cardiac energetic failure and cardiovascular disease [72].

### 4.3. Neuromuscular and Neurodegenerative Disorders

Skeletal muscle is an energy-demanding tissue where defects in mitochondrial respiratory chain proteins can severely compromise function. Consequently, myopathy ranks among the most frequent clinical manifestations of mitochondrial dysfunction (Figure 2, Figure 3, Figure 4, Figure 5, Figure 6 and Figure 7) [74].

#### 4.3.1. Mitochondrial Myopathies

Mitochondrial myopathies (MM) constitute a heterogeneous group of disorders primarily affecting skeletal muscle, characterized by progressive limb weakness, exercise intolerance, and hallmark histological features such as ragged-red and cytochrome c oxidase–deficient fibers. While more than three mt-tRNA^Pro^ mutations have been identified in myopathies, most lack proven pathogenicity. For example, the common A15965G substitution, found in the brain of a patient with idiopathic Parkinson’s disease, is likely benign [75], and the 15 bp duplication m.16018_16032dup in a patient with isolated dilated cardiomyopathy may alter mt-tRNA^Pro^ stability or secondary structure, but clinical significance remains uncertain [76].

Among confirmed pathogenic variants, the G7453A mutation in the acceptor stem of mt-tRNA^Ser(UCN)^ is supported by strong genetic and functional evidence. This mutation reduces tRNA stability, impairs mitochondrial translation, and causes combined deficiencies of complexes I, III, and IV, leading to defective oxidative phosphorylation, increased ROS, and loss of mitochondrial membrane potential. Clinically, it manifests as mitochondrial myopathy and can present with severe neonatal lactic acidosis [77]. More broadly, mt-tRNA^Ser(UCN)^ is among the most frequently disease-associated tRNAs, with at least 16 reported pathogenic variants, often linked to myopathy and deafness [78]. Variants clustering in the acceptor stem, including A7451T, G7453A, and T7510–7512C, underscore the critical role of this region in tRNA processing and mitochondrial function [78].

Another well-established mutation, A15992T, impairs respiratory chain activity, reduces mt-tRNA^Pro^ levels, and causes exercise-induced muscle swelling, intolerance, and hyperlactatemia. It is also associated with lipid accumulation in muscle fibers and early cardiac remodeling, while the related A15992G variant, though affecting the same nucleotide, has less certain pathogenicity [74]. The A3243G mutation in tRNA^Leu^, beyond its classical association with MELAS, can also cause adult-onset mitochondrial myopathy, as illustrated by a case of severe pneumonia with prolonged ventilatory dependence due to respiratory muscle weakness [79]. Additionally, the T14674C mutation in mt-tRNA^Glu^ is linked to reversible infantile respiratory chain deficiency, initially presenting with severe myopathy and hypotonia. This variant may follow a digenic inheritance pattern, requiring an additional heterozygous nuclear mutation for disease manifestation and severity [80].

The genetic spectrum of mitochondrial myopathies extends to numerous additional mt-tRNA variants across multiple tRNA genes in MITOMAP: G5591A, G5610A, G5631A, and G5650A in tRNA^Ala^; G10406A, G10437A, and A10438T in MT-TR; C5708T, T5658C, T5728C, and A5670G in tRNA^Asn^; A7526G in tRNA^Asp^; A4281G in tRNA^Ile^; C3254G, A3243T, A3251G, A3280G, A3288G, and T3273C in tRNA^Leu^; A12320G in tRNA^Leu(CUN)^; C8305T, T8355C, T8362G, and G8340A in tRNA^Lys^; G4403A, G4440A, T4409C, G4450A, and G4412A in tRNA^Met^; T582C, T618C, T618G, and A606G in tRNA^Phe^; 16021_16022delCT, A15998T, T16015C, and A15958T in tRNA^Pro^; G12207A and C12262A in tRNA^Ser(AGY)^; T7480G, A7472C, G7497A, A7472CA, and C7471CC in tRNA^Ser(UCN)^; G5521A, G5522A, T5543C, and T5567C in tRNA^Trp^; and G5835A in tRNA^Tyr^. These mutations collectively demonstrate the critical dependence of skeletal muscle on intact mitochondrial protein synthesis.

#### 4.3.2. Mitochondrial Encephalopathies

Mitochondrial encephalopathies are clinically diverse disorders affecting brain function, often presenting with seizures, cognitive decline, or progressive neurological deterioration. These conditions are frequently caused by pathogenic mitochondrial DNA mutations, particularly in tRNA genes, which disrupt energy metabolism in neurons [81]. tRNA^Asn^ mutations such as T5688C and G5691A lead to epileptic encephalopathy with intellectual disability, seizures, and progressive neurological deterioration. Functional studies demonstrate that these variants destabilize mt-tRNA^Asn^, reduce respiratory chain protein levels, impair oxidative phosphorylation, and trigger excessive mitophagy, highlighting the brain’s particular vulnerability to mitochondrial dysfunction [81]. Similarly, the novel A7484G mutation in mt-tRNA^Ser(UCN)^ was identified in a girl and her mother with severe neurological symptoms, including epilepsy, spastic tetraplegia, and MELAS/MERRF overlap. This mutation disrupts the anticodon region, impairing mitochondrial protein synthesis and respiratory chain function [82]. Encephalopathies associated with mt-tRNA mutations extend beyond the well-characterized syndromes to include numerous variants in MITOMAP: A10438G in MT-TR; T5693C in tRNA^Asn^; A10006G in tRNA^Gly^; T5814C in tRNA^Cys^; G4332A and C4372T in tRNA^Gln^; G14710A, G14739A, T14728C, and C14680A in tRNA^Glu^; T4290C in tRNA^Ile^; C3287A and 3274_3275delAC in tRNA^Leu^; A8302T and G8328A in tRNA^Lys^; A641T and C602T in tRNA^Phe^; G12207A in tRNA^Ser(AGY)^; G15915A in tRNA^Thr^; and G5549A, G5538A, G5540A, G5556C, and G5513A in tRNA^Trp^, collectively highlighting the brain’s exceptional vulnerability to mitochondrial dysfunction.

#### 4.3.3. Parkinson’s Disease and Other Neurodegenerative Disorders

Mitochondrial tRNA mutations also contribute to neurodegenerative and psychiatric disorders. In Parkinson’s disease, variants such as tRNA^Gln^ T4336C, C4335T, and tRNA^Thr^ A15924G, G15927A impair tRNA metabolism, leading to defective translation, reduced ATP synthesis and mitochondrial membrane potential (MMP), and increased ROS production, thereby promoting dopaminergic neurodegeneration [83]. Some of these mutations, such as A15924G, are additionally associated with severe mitochondrial disorders, including lethal infantile myopathy [84].

Alzheimer’s disease (AD), Parkinson’s disease (PD), and multiple sclerosis (MS) are neurodegenerative disorders that share overlapping pathological features and associations with mitochondrial dysfunction. In AD, no confirmed mt-tRNA mutations have been definitively established as causative, but tRNA^Gln^ A4336G has been suggested as a susceptibility factor, observed in approximately 5% of late-onset patients. In PD, mitochondrial tRNA mutations include tRNA^Gln^ A4336G, tRNA^Thr^ G15950A, tRNA^Pro^ T15965C, and tRNA^Thr^ G15927A and G15928A, some of which may contribute to dopaminergic neuron loss, though the association of A4336G with PD is inconsistent [85]. Recently, G15927A tRNA^Thr^ and C4335T tRNA^Gln^ have been proposed to mark early- and late-onset PD, respectively [83]. In MS, tRNA^Thr^ G15927A and G15928A occur more frequently in patients with severe optic dysfunction, but large-scale mtDNA studies have not found pathogenic mutations consistently linked to the disease [86].

AD and PD share overlapping clinical and neuropathological features, including substantia nigra degeneration and Lewy bodies in up to 30% of AD autopsies, while PD patients often develop dementia associated with AD pathology [85]. Mitochondrial dysfunction, particularly complex I inhibition and deficiency, is a central feature of PD pathogenesis [87]. Polymorphic mtDNA variants, including A4336G in tRNA^Gln^, occur at higher frequencies in both disorders compared with controls. Over 30 variants in tRNA^Gln^ have been identified, with G4332A, C4335T, T4336C, and C4349T confirmed pathogenic, producing symptoms ranging from sensorineural deafness and aphasia to Parkinson’s disease and cerebral atrophy. A novel pathogenic variant, T4344C, causes developmental delay and disrupts complexes I, III, and IV [88].

#### 4.3.4. Psychiatric Disorders

In psychiatric disorders such as major depressive disorder (MDD) and other stress-related conditions, the tRNA^Cys^ G5783A mutation has been found in two unrelated families. This mutation is in the structurally critical T-arm stem and destabilizes tRNA function, leading to impaired mitochondrial protein synthesis and widespread respiratory chain deficiencies [89,90]. Clinically, affected individuals exhibited multisystemic features, including myopathy, cardiomyopathy, renal failure, and neurosensory hearing loss, in addition to psychiatric symptoms [91].

### 4.4. Sensory Disorders

Mitochondrial tRNA mutations contribute to a range of sensory disorders by impairing mitochondrial translation, oxidative phosphorylation, and energy metabolism in highly metabolically active tissues such as cochlea, retina, and lens (Figure 2, Figure 3, Figure 4, Figure 5, Figure 6 and Figure 7).

#### 4.4.1. Non-Syndromic Sensorineural Hearing Loss (NSHL)

Mitochondrial tRNA mutations are increasingly implicated in NSHL. Variants in mt-tRNA^Ser(UCN)^, including A7445G and T7510C, reduce tRNA stability and impair translation of COI and ND6, leading to variable hearing loss penetrance and, occasionally, additional neurological or skin phenotypes [92,93,94]. Other pathogenic variants include T593C in tRNA^Phe^, C5783T in tRNA^Cys^, C5601T in tRNA^Ala^, and T12311C in tRNA^Leu(CUN)^, all of which impair tRNA structure, mitochondrial translation, ATP production, and oxidative phosphorylation, leading to cochlear cell apoptosis and hearing loss [95,96,97]. The mutational spectrum of mitochondrial hearing loss extends to over 60 additional variants across multiple mt-tRNA genes in MITOMAP, including T5618C, T5641C, and T5628C in tRNA^Ala^; A7551G and G7566A in tRNA^Asp^; T5802C, T5814C, G5809A, G5822A, T5794C, and G5780A in tRNA^Cys^; A10005G, C10019T, and T10057C in tRNA^Gly^; T12167C, T12188C, T12201C, and G12183A in tRNA^His^; T4268C and A4316G in tRNA^Ile^; C12325T in tRNA^Leu(CUN)^; A8339G and G8340A in tRNA^Lys^; C4437T and A4435G in tRNA^Met^; A636G, C628T, G622A, G586A, and G625A in tRNA^Phe^; T15997C in tRNA^Pro^; G12236A, C12224T, T12235C, C12264T, T12261C, and C12262A in tRNA^Ser(AGY)^; A7456G, C7462T, T7496C, A7474del, A7474G, C7492T, T7505C, C7471del, T7511C, A7445C, and A7445T in tRNA^Ser(UCN)^; T15908C, A15901G, A15902G, C15926T, and G15930A in tRNA^Thr^; and A5568G, G5540A, and A5558G in tRNA^Trp^. These mutations collectively impair tRNA stability, aminoacylation, and mitochondrial translation, leading to cochlear cell dysfunction and progressive hearing loss [97,98].

#### 4.4.2. Leber’s Hereditary Optic Neuropathy

Leber’s hereditary optic neuropathy (LHON) is the most common form of inherited optic neuropathy and mtDNA-related disease, typically presenting in young adults as bilateral, painless, subacute visual failure [99]. Emerging evidence links mt-tRNA mutations to LHON pathogenesis. The mt-tRNA^Glu^ A14693G variant acts as a key modifier, increasing penetrance and severity when present alongside primary complex I mutations such as T14484C. This mutation disrupts the highly conserved pseudouridinylation at position 55, destabilizing tRNA^Glu^ structure and impairing mitochondrial translation. Cells harboring this variant activate compensatory mitophagy to maintain ATP production and reduce ROS; however, in the presence of the primary ND6 mutation, this balance fails, leading to excessive autophagy, apoptosis, and profound respiratory deficiency [100]. A second novel variant, mt-tRNA^Thr^ G15950A, has been identified in LHON patients, expanding the spectrum of mt-tRNA-associated mitochondrial disorders. This mutation impairs mitochondrial translation, particularly of threonine-rich complex I subunits, resulting in OXPHOS dysfunction, elevated ROS, and optic neuropathy [101]. Beyond the primary complex I mutations, additional mt-tRNA variants in MITOMAP may act as disease modifiers or primary pathogenic mutations in LHON, including T5587C in tRNA^Ala^, A4381G and T4363C in tRNA^Gln^, C3275A and C3275T in tRNA^Leu^, A4435G in tRNA^Met^, T593C in tRNA^Phe^, G15986GG in tRNA^Pro^, and A15951G and G15950A in tRNA^Thr^, illustrating the complex genetic architecture of this disorder.

#### 4.4.3. Cataract

Recent studies implicate mt-tRNA mutations in cataract pathogenesis. Cataract, the opacification of the lens, is a leading cause of vision loss worldwide, influenced by age, genetics, ultraviolet exposure, and metabolic conditions such as diabetes [102]. Specifically, mt-tRNA^Ser(AGY)^ mutations C12264T and T12261C have been identified in multisystem mitochondrial disease featuring progressive cataracts [103]. Notably, these mutations were present at low levels in blood but nearly homoplasmic in cataract tissue, indicating tissue-specific enrichment. Molecularly, they disrupt G–C pairing in the tRNA acceptor stem, destabilizing tRNA^Ser(AGY)^, impairing mitochondrial translation, and reducing complex I and IV activity, consistent with respiratory chain dysfunction. These findings illustrate that mt-tRNA defects can compromise the high metabolic demands of the lens, increase susceptibility to oxidative stress, and directly contribute to cataract formation, particularly in the context of systemic mitochondrial disease [104]. In MITOMAP cataracts have been reported with G14685A in tRNA^Glu^, T12148C in tRNA^His^, and A3274G in tRNA^Leu^.

### 4.5. Renal Disorders

Renal tubules, being highly energy-dependent, are particularly vulnerable to mtDNA mutations. Renal involvement in mitochondrial disorders is more frequent in pediatric patients but also affects adults, manifesting as tubular dysfunctions, chronic tubulointerstitial nephritis, cystic kidney disease, and glomerulopathies. Most patients present in the second or third decade, with over half developing chronic kidney disease (CKD). Recurrent myoglobinuria from impaired glycogen or fatty acid metabolism often accompanies tubular injury [105,106].

#### 4.5.1. Glomerular Disease

Two major categories of glomerular disease characterize mitochondrial disorders: mtDNA mutations affecting the tRNA^Leu^ gene and defects in CoQ10 biosynthesis. Focal segmental glomerulosclerosis (FSGS) is the most common histological finding associated with mtDNA point mutations [105]. Although renal manifestations are uncommon in MELAS, carriers of the A3243G mutation frequently develop proteinuria and renal failure, often with diabetes mellitus, sensorineural hearing loss, and neuromuscular deficits. Proteinuric glomerulopathy below nephrotic range affects two-thirds of patients, with FSGS predominating histologically. Up to 80% develop deafness, potentially mimicking Alport syndrome; however, absence of hematuria and ultrastructural glomerular abnormalities distinguishes mitochondrial tRNA^Leu^ mutations from Alport disease. Over 30 MELAS cases with renal involvement have been reported, predominantly in females with onset between 14 and 50 years, half progressing to CKD alongside neurological symptoms [105].

Proteomic analyses of 117 adult mitochondrial disease patients revealed that 75 carried A3243G, with nearly half exhibiting albuminuria or low-molecular-weight proteinuria. Approximately half presented with maternally inherited diabetes and deafness (MIDD), while others manifested MELAS or MERRF [107]. Other rare mtDNA point mutations (A4269G, A5728G, A5843G) are occasionally associated with FSGS. While mtDNA deletions in Kearns–Sayre and Pearson syndromes seldom cause glomerular disease, they commonly link to tubular dysfunction and interstitial nephritis. A novel G5538A variant in tRNA^Trp^ was recently reported in FSGS with multisystem involvement, expanding the mitochondrial nephropathy genotype-phenotype spectrum [106]. Additional mt-tRNA variants associated with focal segmental glomerulosclerosis include T7560C in tRNA^Asp^ and A5843G in tRNA^Tyr^, expanding the genetic spectrum beyond the classic A3243G mutation [106].

#### 4.5.2. Mitochondrial Tubulointerstitial Kidney Disease (MITKD)

The concept of mitochondrial tubulointerstitial kidney disease has emerged to describe renal pathology directly caused by mtDNA mutations. A notable example is T616C in mt-tRNA^Phe^, identified in heteroplasmic and homoplasmic states in families with CKD and hyperuricemia. Homoplasmic carriers often develop multisystem disease, including epilepsy, encephalopathy, and progressive renal failure. Functionally, T616C disrupts the conserved A31-U39 base pair in mt-tRNA^Phe^’s anticodon stem, destabilizing structure, reducing steady-state levels by approximately 50%, and impairing aminoacylation. This causes defective mitochondrial translation and reduced expression of multiple OXPHOS subunits (ND1–6, ND4L, CYTB, CO1–3, ATP6, ATP8). Compensatory mechanisms, including enhanced aminoacylation and increased mitophagy, may explain the high pathogenicity threshold observed clinically. The T616C variant illustrates how mt-tRNA instability disrupts mitochondrial protein synthesis and contributes to CKD pathogenesis [108]. Beyond T616C in tRNA^Phe^, additional nephropathy-associated variants in MITOMAP include 3274_3275delAC and G3242A in tRNA^Leu^, A608G in tRNA^Phe^, and T7501A in tRNA^Ser(UCN)^, demonstrating the kidney’s vulnerability to mitochondrial tRNA defects.

Gitelman-like syndrome, characterized by hypomagnesemic metabolic syndrome, has been linked to T4291C in tRNA^Ile^, A643G and C591T in tRNA^Phe^, and T616C in tRNA^Phe^ (also causing maternally inherited epilepsy and mitochondrial tubulointerstitial kidney disease), suggesting shared pathogenic mechanisms involving renal tubular ion transport.

### 4.6. Metabolic and Endocrine Disorders

Endocrine abnormalities are frequent in inherited mitochondrial disorders and often arise from impaired intracellular hormone synthesis or reduced extracellular secretion. Diabetes mellitus is the most common manifestation, although growth hormone deficiency, hypogonadism, adrenal dysfunction, hypoparathyroidism, and thyroid disease are also reported [109]. While endocrine dysfunction typically occurs as part of multisystem involvement, certain mitochondrial disorders may present primarily with isolated endocrine phenotypes (Figure 2, Figure 3, Figure 4, Figure 5, Figure 6 and Figure 7).

#### 4.6.1. Diabetes Mellitus

Diabetes mellitus, a central metabolic disease associated with cardiovascular disease, stroke, hypertension, and deafness, is characterized by persistent hyperglycemia due to impaired insulin secretion, insulin resistance, or both [14]. Diabetes has been reported in chronic progressive external ophthalmoplegia, Kearns–Sayre syndrome, Pearson syndrome, MELAS, and less commonly in MERRF. The A3243G mutation in the mitochondrial tRNA^Leu(UUR)^ gene is a key molecular determinant, accounting for approximately 1.5% of diabetes cases worldwide. Clinically, A3243G heteroplasmy correlates with diabetes incidence, earlier onset, and intergenerational disease burden [110]. Diabetes associated with this mutation reflects primarily impaired β-cell secretion, characterized by reduced insulin and C-peptide responses, decreased urinary C-peptide excretion, and minimal insulin resistance [108,110].

Other mutations in tRNA^Leu(UUR)^ and elsewhere in mtDNA also influence diabetes susceptibility [110,111]. Variants in tRNA^Ile^, tRNA^Ser(UCN)^, tRNA^Lys^, MT-ND1, MT-ND4, MT-COX2, and MT-COX3 have been identified, including T3264C (tRNA^Leu(UUR)^), T4291C (tRNA^Ile^), T14709C and A14692G (tRNA^Glu^), and T10003C (tRNA^Gly^), T3271C (tRNA^Leu^), C3254A (tRNA^Leu^), and A8344G (tRNA^Lys^), all linked to type 2 diabetes. The G15897A mutation in tRNA^Thr^ is associated with maternally transmitted T2DM, with structural modeling suggesting impaired aminoacylation [112]. Other reported variants include tRNA^Trp^ A5514G and tRNA^Ser(AGY)^ C12237T, reflecting heteroplasmy and phenotypic variability. A novel A14687G tRNA^Glu^ variant was recently identified as a potential T2DM risk factor [113].

Overall, the mitochondrial tRNA^Leu(UUR)^ gene represents a mutational hotspot, accounting for approximately 60% of reported disease-associated mt-tRNA mutations. Rare substitutions within this gene have been linked to diabetes in combination with neuromuscular and endocrine phenotypes, including encephalomyopathy, pigmentary retinopathy, and hypothyroidism [110]. Mutations at positions 3252, 3254, 3256, and 3260 have been associated with multisystemic disorders, further underscoring the role of base substitutions in this locus in mitochondrial disease pathogenesis. Beyond A3243G, additional mt-tRNA mutations associated with diabetes include T3264C in tRNA^Leu^ (diabetes mellitus) and C3254A (gestational diabetes), T12317C in tRNA^Leu(CUN)^ and T12278C, T10003C in tRNA^Gly^, and T4289C in tRNA^Ile^, highlighting the role of mitochondrial dysfunction in pancreatic β-cell failure and insulin secretion [110,114].

#### 4.6.2. Polycystic Ovary Syndrome (PCOS)

PCOS is the most prevalent endocrine disorder, affecting up to 15% of women of reproductive age worldwide. It is clinically defined by chronic anovulation, hyperandrogenism, and the presence of polycystic ovaries [115]. Mitochondria play a central role in oxidative stress, which is increasingly recognized as a key factor in PCOS pathogenesis [115]. Meta-analytical studies have consistently shown that mutations have been identified in oxidative phosphorylation (OXPHOS) subunits as well as in mitochondrial tRNA (mt-tRNA) genes, implicating mitochondrial dysfunction as a pathogenic driver [116]. To date, 12 mutations have been identified within eight distinct mt-tRNA genes, underscoring the contribution of tRNA dysfunction to PCOS development [117].

Nine mt-tRNA mutations have been associated with insulin resistance in PCOS, including mt-tRNA^Leu(UUR)^ (A3302G, C3275A), mt-tRNA^Gln^ (T4363C, T4395C), mt-tRNA^Ser(UCN)^ (C7492T), mt-tRNA^Asp^ (A7543G), mt-tRNA^Lys^ (A8343G), mt-tRNA^Arg^ (T10454C), and mt-tRNA^Glu^ (A14693G) [118]. These variants destabilize tRNA secondary and tertiary structures, reducing aminoacylation efficiency, steady state tRNA levels, and codon recognition. Functionally, they lead to increased ROS, decreased mitochondrial membrane potential (MMP), reduced ATP production, and lower mtDNA copy numbers, all of which contribute to PCOS-associated metabolic dysfunction [116]. The mt-tRNA^Ser(UCN)^ C7492T mutation, located at a highly conserved site within the anticodon stem, disrupts aminoacylation and tRNA stability, mechanistically linking it to mitochondrial dysfunction and PCOS pathogenesis [115]. The causal role of mt-tRNA mutations is further supported by animal studies: a transgenic mouse harboring the A3302G mutation in mt-tRNA^Leu(UUR)^ exhibited metabolic syndrome features [117].

Additional mt-tRNA loci are implicated in PCOS. The tRNA^Leu(CUN)^ gene harbors three mutations (C12267T, A12307G, A12308G), with A12308G repeatedly identified in PCOS cohorts. Variants in tRNA^Ser(UCN)^ and tRNA^Thr^ have also been reported. tRNA^Ser(UCN)^ encodes a serine tRNA and harbors the A7490G mutation in the anticodon stem, likely destabilizing tRNA folding and aminoacylation. tRNA^Thr^ includes G15928A associated with PCOS, multiple sclerosis, and idiopathic recurrent miscarriage. Additional tRNA^Thr^ variants (A15914T, A15924G, G15930A) identified in Indian cohorts suggest possible population-specific mutation spectra. Despite minimal changes in minimum free energy, clustering within functionally critical regions supports tRNA^Thr^ involvement in PCOS. Variants in tRNA^His^ (A12179T, A12182G) may alter histidine incorporation during mitochondrial translation, impairing energy metabolism [117].

Beyond point mutations, large-scale mtDNA deletions also contribute to PCOS. The “common deletion” of 4977 bp removes about one-third of the mitochondrial genome, including five tRNA genes and seven OXPHOS subunits (complexes I, IV, and V). Originating from replication slippage and accumulating with age, this deletion is linked to type 2 diabetes mellitus, aging, and ovarian senescence. While not PCOS-specific, evidence suggests it may contribute to disease progression in a subset of patients [118]. Additional variants implicated in PCOS pathogenesis in MITOMAP include T4363C in tRNA^Gln^ and C3275T in tRNA^Leu^, further supporting the role of mitochondrial tRNA dysfunction in ovarian and metabolic pathology.

#### 4.6.3. Autoimmune Endocrinopathies

Mitochondrial dysfunction has also been proposed in the context of autoimmune endocrinopathies. Autoimmune polyendocrinopathy type II (APS2) was reported in a single patient with Kearns–Sayre syndrome carrying two maternally transmitted mutations: one in mt-tRNA^Met^ (A4415G) and another in COXIII (A9922C). While the pathogenic mechanisms remain speculative, recent studies suggest that mitochondrial tRNA^Met^ exported to the cytoplasm is required for Argonaute 2 (Ago2) activity, the catalytic core of the RNA-induced silencing complex (RISC). Disruption of this interaction by the A4415G mutation may impair self/non-self RNA discrimination and thereby trigger autoimmune responses, offering a novel mechanistic link between mitochondrial dysfunction and immune dysregulation [119].

### 4.7. Other Disorders

Although mitochondrial dysfunction is increasingly recognized in hematologic disorders, there are very few reports in which a specific mutation in a mitochondrial tRNA gene directly causes a hematologic disease. Most hematologic involvement in mitochondrial syndromes arises secondarily to broader systemic defects (Figure 2, Figure 3, Figure 4, Figure 5, Figure 6 and Figure 7).

#### 4.7.1. Myelodysplastic Syndrome (MDS)

One notable variant is the G3242A mutation in the mitochondrial tRNA^Leu(UUR)^ gene, which was identified in the bone marrow of a patient with MDS and ineffective hematopoiesis. The mutation was confined to hematopoietic progenitor cells, particularly CD34^+^ stem cells, but was strongly selected against during differentiation, explaining its absence in mature blood lineages. This suggests that while hematopoietic stem cells can tolerate impaired mitochondrial translation through glycolysis, differentiating progenitors—especially erythroid cells that depend on mitochondrial respiratory function for heme synthesis—are highly vulnerable. Although this mutation’s causal role requires further confirmation, it may directly contribute to bone marrow failure syndromes such as MDS, linking defective mitochondrial protein synthesis to impaired hematopoiesis [120].

#### 4.7.2. Systemic Lupus Erythematosus (SLE)

SLE, an autoimmune-mediated multisystem disease predominantly affecting women of reproductive age, involves a complex interplay of genetic, epigenetic, environmental, and mitochondrial factors. Screening for mt-tRNA variants in SLE has identified several candidates—tRNA^Val^ G1606A, tRNA^Leu(UUR)^ A3243G, tRNA^Ile^ A4295G, tRNA^Gly^ T9997C, and tRNA^Thr^ A15924G—that may alter tRNA folding, aminoacylation, or codon recognition. Functional studies have demonstrated that these mutations impair mitochondrial translation, reduce OXPHOS complex activity, and contribute to cellular energy failure, thereby offering new mechanistic insight into the contribution of mitochondrial genetics to autoimmune disease [121].

#### 4.7.3. Tic Disorders (TD)

TD are neuropsychiatric conditions characterized by repetitive, involuntary movements or vocalizations, affecting up to 25% of children, with higher prevalence in males. The DSM-V classifies TD into Tourette’s syndrome (TS), chronic tic disorder (CTD), and provisional tic disorder (PTD). Although TD is genetically complex, the contribution of mitochondrial tRNA variants remains underexplored. Several mtDNA mutations may alter tRNA structure, aminoacylation efficiency, and oxidative phosphorylation, contributing to TD pathogenesis. The A5587G variant at the 3′ terminus of tRNA^Ala^ may disrupt tRNA folding and amino acid incorporation, impairing mitochondrial respiratory chain function. Alterations such as A5558G and G5595A in the T-stem of tRNA^Trp^, and A4317G in tRNA^Ile^—previously linked to cardiomyopathy—can impair tRNA processing, stability, and CCA addition. Variants, including A10055G (tRNA^Gly^), A12141G, A15948G, G15950A, and C15952T (tRNA^His^), disrupt acceptor-stem base pairing, reducing ATP production and membrane potential, while C15910T in tRNA^Thr^ and T4373C in tRNA^Gln^ destabilize their respective stems. Variants in the D- or T-loops (e.g., tRNA^Ser(UCN)^ C7502T, tRNA^Leu(CUN)^ A12279G) impair tertiary structure and aminoacyl-tRNA synthetase recognition. Mutations such as T5794C and A15992G disrupt anticodon modification and codon recognition, while A14692G, T4452C, T5819C, and A7526G compromise tRNA stability and aminoacylation, contributing to mitochondrial dysfunction [122]. Additional mt-tRNA variants identified in tic disorder patients in MITOMAP include G5595A and T5628C in tRNA^Ala^, T5587C in tRNA^Ala^, T5794C and T5774C in tRNA^Cys^, T4373C in tRNA^Gln^, A14692G in tRNA^Glu^, A10055G in tRNA^Gly^, A12141G in tRNA^His^, A12279G in tRNA^Leu(CUN)^, C7502T in tRNA^Ser(UCN)^, G15950A in tRNA^Thr^, and A5558G in tRNA^Trp^, further implicating mitochondrial dysfunction in the neurobiology of Tourette’s syndrome and related disorders.

#### 4.7.4. Autism Spectrum Disorders (ASD)

ASD are early-onset developmental conditions with multifactorial etiology involving over 1000 genes and environmental factors, including prenatal toxin exposure, immune activation, and metabolic disturbances [123]. Mitochondrial dysfunction is common in ASD and may arise from mtDNA alterations or external stressors [124]. The G8363A variant in mtDNA tRNA^Lys^, initially linked to encephalomyopathy, sensorineural hearing loss, and hypertrophic cardiomyopathy, has been associated with autism, Leigh syndrome, MERRF, ataxia, and lipomas [125]. Beyond G8363A, additional ASD-associated variants include T4336C in tRNA^Gln^, A4388G in tRNA^Gln^, A14687G in tRNA^Glu^, A4435G in tRNA^Met^, and C12264T in tRNA^Ser(AGY)^, underscoring the heterogeneity of mitochondrial autism. Screening studies identified A3243G in only 0.2% of ASD cases, while others found no association with mtDNA haplogroups [126]. Rare pathogenic variants such as A3397G (ND1), A4295G (tRNA^Ile^), and T11984C (ND4) affect mitochondrial function, and variants like T15067C, C15202T, and T15262C correlate with elevated lactate, while C14753T and T15262C are linked to increased ammonia levels [127]. These findings highlight the heterogeneity of ASD associated with mitochondrial oxidative phosphorylation defects, distinct from idiopathic autism, while suggesting a broader spectrum of “mitochondrial autism” [128].

#### 4.7.5. Cancer

Emerging evidence increasingly supports a role for mitochondrial tRNA mutations in carcinogenesis through disruption of mitochondrial translation and oxidative phosphorylation. Pathogenic variants in mt-tRNA genes, particularly in tRNA^Leu(UUR)^ and tRNA^Lys^, have been recurrently identified in breast and other solid tumors [129]. Among these, the well-characterized A3243G mutation in tRNA^Leu(UUR)^, has been reported in invasive breast carcinoma and other malignancies, often co-occurring with impaired complex I activity and increased reactive oxygen species production [130]. Similarly, the A8344G substitution in tRNA^Lys^, a variant classically associated with MERRF, has also been detected in tumor tissues, suggesting that such pathogenic variants may be maintained in somatic or germline lineages and contribute to altered bioenergetics and enhanced tumor growth [131]. In addition to these well-known variants, recent sequencing studies have identified other potentially pathogenic mutations in mt-tRNA genes among cancer patients, including tRNA^Val^ G1606A, tRNA^Ile^ A4300G, tRNA^Ser(UCN)^ T7505C, tRNA^Glu^ A14693G, and tRNA^Thr^ G15927A [132]. These mutations occur at evolutionarily conserved nucleotides and are predicted to destabilize tRNA secondary structure, interfere with aminoacylation or post-transcriptional modification, and ultimately impair mitochondrial translation efficiency. The resulting dysfunction of oxidative phosphorylation (OXPHOS), ATP depletion, and excessive ROS generation create a metabolic environment favorable for tumorigenesis. Collectively, these findings indicate that mt-tRNA point mutations, especially those located in key structural domains such as the D-loop, anticodon stem, or T-arm, represent an underrecognized but mechanistically relevant class of mitochondrial genome alterations contributing to cancer development and progression [132].

#### 4.7.6. Additional Rare Phenotypes

A case of mitochondrial encephalomyopathy associated with tRNA^Val^ A1630G presented primarily with epilepsy [133]. Pathogenic variants in tRNA^Asn^ are exceedingly rare, with only five reported, causing phenotypes that range from chronic progressive external ophthalmoplegia to lethal neonatal encephalomyopathy. A novel T5709C variant further broadens this spectrum, linking tRNA^Asn^ dysfunction to ophthalmoparesis and respiratory failure [134]. Additional associations in MITOMAP include C8346del in tRNA^Lys^ with Rett syndrome; A1661G and A1636G in tRNA^Val^ with Charcot–Marie–Tooth disease featuring global developmental delay, progressive myoclonic epilepsy, paroxysmal arrhythmia, and brain atrophy; A10044G in tRNA^Gly^ with sudden infant death syndrome; and T8357C in tRNA^Lys^ with multiple symmetric lipomatosis and multiorgan disease (Figure 2, Figure 3, Figure 4, Figure 5, Figure 6 and Figure 7).

## 5. Post-Transcriptional Modifications of Mitochondrial tRNAs and Disease Pathogenesis

In addition to pathogenic mutations within mitochondrial tRNA genes discussed above, defects in post-transcriptional modification systems represent an increasingly recognized mechanism of mitochondrial disease. While the preceding sections have extensively characterized pathogenic mutations within mt-tRNA genes, the enzymatic machinery responsible for chemical modification of these tRNAs after transcription constitutes an equally critical layer of regulation. Disruption of these modification processes-either through mutations in nuclear-encoded modifying enzymes or indirectly through mt-tRNA structural variants that prevent enzyme recognition-can provoke severe mitochondrial dysfunction even in the absence of primary mt-tRNA sequence alterations.

### 5.1. The Landscape of Mitochondrial tRNA Modifications

Mitochondrial tRNA molecules undergo comprehensive chemical alterations following transcription, which prove critical for maintaining their structural integrity, enabling precise codon recognition, optimizing translational efficiency, and conferring resistance to nuclease-mediated degradation. In contrast to nuclear-encoded cytoplasmic tRNAs that harbor more than 100 distinct modification types, mitochondrial tRNAs exhibit a more restricted yet functionally essential repertoire of modifications. This distinction reflects the evolutionary constraints and specialized requirements inherent to the mitochondrial translation machinery. These chemical modifications are introduced at defined nucleotide positions through the catalytic activity of nuclear-encoded modifying enzymes, which must undergo import into mitochondria, thereby establishing an indispensable nuclear-mitochondrial coordination mechanism for preserving mt-tRNA functionality [135]. Recent advances in analytical methodologies have significantly expanded our ability to characterize mitochondrial tRNA modifications. Techniques such as liquid chromatography–tandem mass spectrometry (LC-MS/MS), high-throughput RNA modification mapping, and nanopore direct RNA sequencing enable precise detection, quantification, and positional assignment of modified nucleotides within mitochondrial tRNAs. These approaches have been instrumental in revealing the diversity of mt-tRNA modification landscapes and in linking specific chemical changes to functional or pathological outcomes [136,137].

Human mitochondrial tRNAs comprise 22 distinct species that collectively contain 18 different chemically modified nucleotides distributed across 137 positions. This means that, on average, each mt-tRNA molecule contains multiple modified nucleotides at specific positions critical for its function. These modified residues constitute approximately 8.7% of the total nucleotide content within mt-tRNAs. Notably, six of these modifications are exclusively present in mitochondrial tRNAs and absent from their cytoplasmic counterparts. Every tRNA modification is introduced post-transcriptionally at designated positions by specific mt-tRNA modification enzymes. Current knowledge identifies 22 mt-tRNA modification enzymes or their associated partner proteins, with estimates suggesting approximately 10 additional modifying enzymes remain to be discovered [13].

### 5.2. Pathogenic Mutations in Modification Enzymes

**Figure 8:**
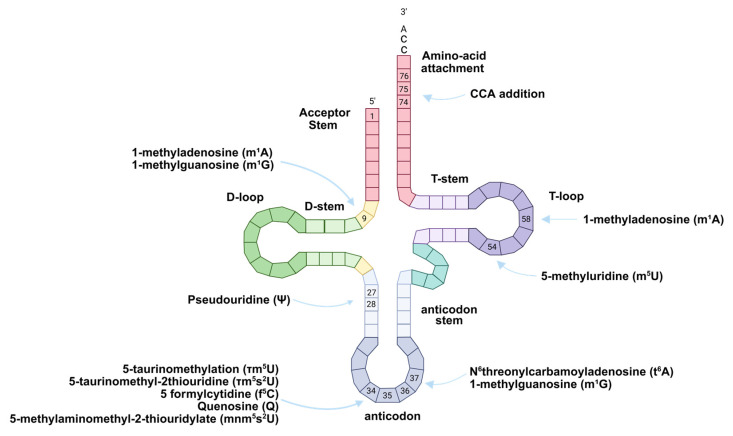
Representative mt-tRNA cloverleaf structure showing the distribution of key post-transcriptional modifications. Essential modifications include pseudouridine (Ψ) at positions 27–28, 5-methyluridine (m^5^U) at position 54, 1-methyladenosine (m^1^A) at position 9, 58, and critical anticodon wobble modifications (position 34) such as τm^5^U, τm^5^s^2^U, f^5^C, Q, and mnm^5^s^2^U. These modifications, introduced by nuclear-encoded enzymes which are essential for tRNA stability, codon recognition, and translational accuracy. Loss of these modifications through enzyme mutations or mt-tRNA sequence variants disrupts mitochondrial translation and causes diseases, including MELAS, MERRF, RILF (reversible infantile liver failure), and MLASA. (Created in BioRender. https://BioRender.com/dufc0i4 (accessed on 12 November 2025)).

As these chemical modifications are essential for mt-tRNA function, pathogenic mutations affecting the modification machinery represent a major causative factor in mitochondrial diseases. Essential modifications, including 5-taurinomethyluridine (τm^5^U), 5-formylcytidine (f^5^C), N6-threonylcarbamoyladenosine (t^6^A), and queuosine (Q), particularly at the anticodon wobble position, are absolutely required for maintaining mt-tRNA stability and ensuring translational fidelity (Figure 8) [135].

To date, pathogenic mutations have been documented in 13 proteins responsible for mt-tRNA modifications: TRMT10C, HSD17B10, TRMT1, PUS1, MTO1, GTPBP3, MTU1, NSUN3, TRMT5, YRDC, TRIT1, NSUN2, and TRMT2B. Among these, eight proteins-namely PUS1, MTO1, GTPBP3, MTU1, NSUN3, TRMT5, TRIT1, and TRMT2Bare directly implicated in mitochondrial diseases based on clinical manifestations and subcellular localization patterns. Interestingly, mutations in TRMT1, YRDC, or NSUN2 appear to cause disease through loss of cytoplasmic tRNA modifications, as these proteins function in both compartments. Additionally, TRMT10C and HSD17B10 form a complex that introduces m1G or m1A at position nine but also constitute components of mitochondrial RNaseP, which processes the 5′ termini of nascent mt-tRNAs, consequently, distinguishing between effects of reduced modification versus impaired RNA processing on disease onset remains challenging [13].

The consequences of losing critical mt-tRNA modifications are profound and multifaceted. Loss of these modifications results in decreased oxidative phosphorylation capacity, leading to compensatory increases in glycolysis to maintain ATP levels. This metabolic shift causes lactate and NADH accumulation, NAD+ depletion, and elevated reactive oxygen species generation, potentially triggering inflammation and cell death. In certain cases, deficient mt-tRNA modifications impair mitochondrial protein import, causing cytoplasmic accumulation and aggregation of mitochondrial proteins that activate the unfolded protein response. Since ATP synthesis is particularly crucial for energy-demanding tissues, including the brain and the heart, loss of important tRNA modifications most frequently manifests as encephalopathy and cardiomyopathy, resulting in severe disability and reduced life expectancy [13].

### 5.3. Modification Defects in Typical Mitochondrial Syndromes

The interplay between mt-tRNA sequence variants and post-transcriptional modification defects is exemplified in several well-characterized mitochondrial syndromes. Defective τm^5^U formation caused by mtDNA mutations (A3243G, U3271C, G3244A, U3258C, U3291C) in mt-tRNA^Leu(UUR)^ prevents recognition by the GTPBP3-MTO1 complex, resulting in MELAS with severe τm^5^U impairment [138]. The mechanism underlying this disease is particularly instructive: hypomodified mt-tRNA^Leu(UUR)^ retains the capacity to decode UUA codons but fails to efficiently decode UUG codons, causing mitoribosome stalling. Since ND6, a complex I subunit, has the highest UUG codon frequency among mtDNA-encoded genes, its translation is severely compromised, thereby explaining the characteristic complex I deficiency observed in MELAS [139].

Similarly, MERRF arises from the A8344G mutation in mt-tRNA^Lys^. This mutation causes loss of τm^5^s^2^U modification, preventing efficient decoding of AAA and AAG codons and resulting in defective mitochondrial translation. Additionally, this mutation causes loss of m1A at position 58, normally incorporated by TRMT61B [139]. Expanding beyond mtDNA mutations, defects in the nuclear-encoded modifying enzymes themselves can also precipitate disease. MTO1 mutations cause combined oxidative phosphorylation deficiency 10 (COXPD10), presenting with infantile hypertrophic cardiomyopathy and lactic acidosis, while GTPBP3 mutations cause COXPD23, characterized by early childhood hypertrophic cardiomyopathy and neurological symptoms [140].

### 5.4. Enzyme-Specific Disease Phenotypes

MTU1, which encodes a mitochondrial 2-thiouridylase, when mutated causes reversible infantile liver failure (RILF) within the first year of life, occasionally accompanied by cardiomyopathy [141]. What makes this condition particularly intriguing is its transient nature–most patients who survive the acute phase recover completely, though the mechanistic basis for this reversibility remains unclear. Functional studies of 16 pathogenic variants revealed markedly decreased protein levels due to CLPX-CLPP protease degradation. Three variants completely lost activity, while 13 retained partial function when protein levels were restored. Critically, patient survival requires ≥10% residual 2-thiolation activity; variants causing both RILF and cardiomyopathy show lower activity levels [142]. In a related manner, TRMU (mitochondrial tRNA 5-methylaminomethyl-2-thiouridylate methyltransferase) mutations cause similar transient infantile liver failure with hepatic involvement, lactic acidosis, emesis/diarrhea, nervous system involvement, and hypotonia [143].

The spectrum of modification-associated diseases extends to include hematological manifestations as well. PUS1, which catalyzes pseudouridine (Ψ) formation at positions 27 and 28, provides an illustrative example. Pseudouridine is a C-C glycosidic isomer of uridine that adopts C3′-endo conformation and enhances base-stacking, conferring higher RNA melting temperatures. When PUS1 is mutated, it causes myopathy, lactic acidosis, and sideroblastic anemia (MLASA), likely through destabilization of anticodon stem helical structure [13]. Another enzyme involved in hematological disease is TRNT1, which encodes tRNA-nucleotidyltransferase that adds CCA trinucleotides to all mt-tRNA and cytoplasmic tRNA 3′ termini, essential for aminoacylation and ribosomal positioning. Seventeen pathogenic variants cause congenital sideroblastic anemias (CSAs) with mitochondrial iron deposition in red blood cell precursors, presenting with retinitis pigmentosa, microcytosis, sideroblastic anemia, B-cell immunodeficiency, developmental delay, and periodic fevers [140].

More recently, mt-tRNA modification defects have been linked to neurodegenerative conditions. TRMT2B, which installs m^5^U at mt-tRNA position 54 and in mt-12S rRNA, has been implicated in amyotrophic lateral sclerosis (ALS). Mutations identified in ALS patients show decreased NADH dehydrogenase subunit 1 levels (complex I), diminished complex I activity, reduced mitochondrial respiration, and elevated ROS. However, the relative contributions of mt-tRNA versus mt-rRNA m^5^U loss to ALS pathogenesis require further investigation via mitoribosome profiling [13]. Additionally, TRMT5, which methylates G37 (m^1^G) in cytoplasmic tRNA^Leu(GAC)^ and mitochondrial tRNA^Pro^, thereby preventing ribosomal frameshift errors, when mutated causes peripheral neuropathy with variable spasticity, exercise intolerance, and developmental delay (PNSED). Eight patients identified with this condition present with hypotonia, developmental delay, peripheral neuropathy, and variable complex I, III, and IV deficiencies [140].

Finally, queuosine (Q) modification at position 34 is essential for decoding Asn, Tyr, His, and Asp codons. Queuine, the substrate for Q modification, regulates translation elongation and enhances mitochondrial mRNA translation efficiency. Q deficiency links to mitochondrial dysfunction and may influence disease severity in mt-tRNA^Asn^ variants. Structural abnormalities in mt-tRNA^Asn^ variants increase susceptibility to ribonuclease cleavage, generating aberrant tRNA-derived stress-induced fragments (tRFs) that impede mtDNA transcription [135].

## 6. Discussion

**Figure 9:**
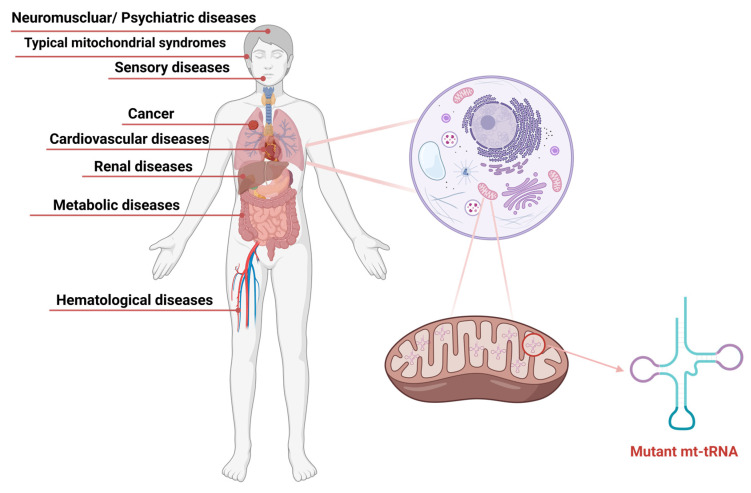
Schematic representation of the multi-organ involvement in mt-tRNA-related diseases. Colored markers indicate affected organ systems. This figure illustrates the remarkable clinical heterogeneity arising from mt-tRNA mutations and emphasizes the importance of comprehensive multi-system evaluation in suspected mitochondrial disease. (Created in BioRender. https://BioRender.com/rz4on29 (accessed on 12 November 2025)).

Pathogenic mutations in mitochondrial tRNA genes represent a critically important yet historically underappreciated category of human disease. Despite constituting only approximately 5–10% of the mitochondrial genome, mt-tRNA genes harbor nearly 70–75% of all pathogenic mtDNA mutations, underscoring their disproportionate contribution to mitochondrial dysfunction and human pathology. This comprehensive systematic review consolidates the current state of knowledge regarding mt-tRNA mutations and their remarkably diverse clinical manifestations, ranging from typical mitochondrial syndromes such as MELAS, MERRF, and Leigh syndrome to an expanding spectrum of organ-specific disorders affecting the cardiovascular, neuromuscular, sensory, renal, metabolic, and hematological systems (Figure 9). This systematic review distinguishes itself from prior literature in three key aspects. First, whereas previous reviews typically organize mt-tRNA mutations either by individual genes or by syndrome, we provide an integrated framework that combines pathogenic variant cataloging with phenotypic system-level classification. Second, we incorporate post-transcriptional modification defects alongside primary sequence mutations, expanding the mechanistic landscape of mt-tRNA-related disease. Third, by aggregating all clinically relevant variants reported through October 2025 and organizing them by affected organ systems, molecular mechanisms, and disease clusters, this work offers a uniquely comprehensive and clinically oriented resource for diagnosticians and genetic counselors.

The clinical heterogeneity associated with mt-tRNA mutations presents substantial diagnostic challenges. A single mutation, such as the A3243G variant in tRNA^Leu^, can manifest across a broad phenotypic continuum—from isolated cardiomyopathy or diabetes mellitus to severe multisystem involvement with stroke-like episodes, hearing loss, and encephalopathy. Conversely, clinically indistinguishable phenotypes may arise from mutations in entirely different mt-tRNA genes, reflecting the complex interplay of tissue-specific energy demands, nuclear-mitochondrial genetic interactions, and environmental modifiers. This genotype-phenotype variability not only complicates clinical diagnosis and genetic counseling but also highlights the urgent need for comprehensive molecular screening strategies and functional validation of novel variants.

**Figure 10:**
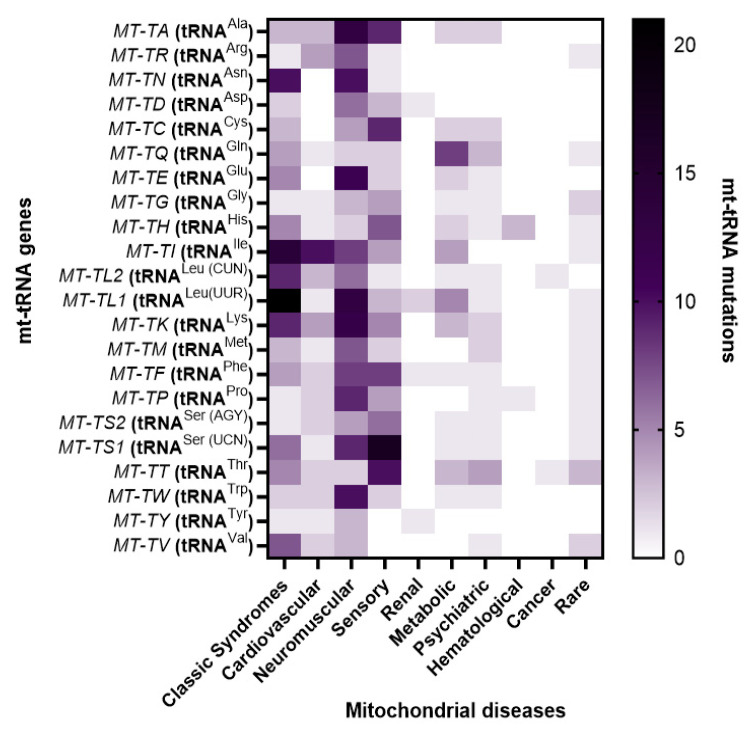
This comprehensive heatmap illustrates the distribution and frequency of reported pathogenic mutations in all 22 mitochondrial tRNA genes (*y*-axis) across 10 major organ systems and disease categories (*x*-axis). Color intensity represents the approximate number of distinct pathogenic variants reported in the MITOMAP database and literature (accessed on 1 October 2025) for each gene-disease combination. Color scale: White = no reported mutations (0), Light purple = 1–5 mutations, Medium purple = 6–10 mutations; Dark purple ≥ 10 mutations. Each cell’s color reflects the cumulative number of pathogenic variants identified for that specific mt-tRNA gene associated with that disease category. All axes include complete and explicit labels: the *x*-axis lists mt-tRNA genes by standard nomenclature (e.g., MT-TL1, MT-TK, MT-TS1, MT-TS2), while the *y*-axis lists major disease categories, including mitochondrial syndromes (MELAS, MERRF, Leigh, etc.), cardiovascular disease, neuromuscular disorders, sensory disorders, renal disease, metabolic/endocrine disorders, psychiatric/neurodevelopmental disorders, and cancer. Gene names and disease categories correspond directly to classifications detailed in the text. This visualization underscores the remarkable clinical heterogeneity of mt-tRNA mutations and highlights gene-specific disease predispositions. Notably, tRNA^Leu(UUR)^, tRNA^Lys^, and tRNA^Ser(UCN)^ harbor the highest mutational burden, while cardiovascular and neuromuscular systems demonstrate the greatest vulnerability across multiple genes. The clustering patterns may guide targeted diagnostic screening strategies based on clinical presentation.

To facilitate clinical interpretation and diagnostic prioritization, we systematically analyzed the distribution of pathogenic mt-tRNA mutations across organ systems. To systematically visualize the distribution of pathogenic mutations, we quantified the number of distinct variants reported for each mt-tRNA gene across major disease categories. This analysis reveals distinct mutational hotspots and tissue-specific vulnerabilities that may guide diagnostic strategies. As illustrated in Figure 10, distinct mutational hotspots emerge among the 22 mt-tRNA genes, with tRNA^Leu^, tRNA^Lys^, and tRNA^Ser(UCN)^ exhibiting the highest burden of reported pathogenic variants. Notably, cardiovascular and neuromuscular systems demonstrate the greatest vulnerability to mt-tRNA dysfunction across multiple genes, consistent with the high energetic demands of these tissues. This comprehensive mapping reveals gene-specific disease predispositions—for instance, tRNA^Ser(UCN)^ mutations are disproportionately associated with sensory disorders, particularly hearing loss, while tRNA^Leu^ variants manifest predominantly in typical mitochondrial syndromes and cardiomyopathies. These patterns provide a framework for targeted genetic screening based on clinical presentation and inform genotype-phenotype correlation studies.

From a mechanistic perspective, mt-tRNA mutations exert their pathogenic effects through diverse molecular pathways. Structural destabilization of the cloverleaf secondary structure, impaired aminoacylation efficiency, disrupted post-transcriptional modifications—particularly at critical wobble positions—altered codon recognition fidelity, and increased susceptibility to degradation collectively converge to impair mitochondrial translation. The resulting deficiencies in respiratory chain complex activity trigger a cascade of cellular dysfunction characterized by ATP depletion, excessive reactive oxygen species production, disrupted calcium homeostasis, and ultimately cell death in high-energy-demand tissues such as neurons, cardiomyocytes, cochlear hair cells, and renal tubular epithelium.

The therapeutic landscape for mt-tRNA-associated disorders has evolved considerably in recent years, driven by advances in understanding post-transcriptional modification defects and their central role in disease pathogenesis. As detailed in Section 5, loss of critical modifications such as τm^5^U, τm^5^s^2^U, and m^1^A profoundly disrupts mitochondrial translation and respiratory chain function, establishing modification enzyme dysfunction as a key therapeutic target. Overexpression of modification enzymes has demonstrated promising efficacy in patient-derived cellular models. In MELAS cybrids harboring the A3243G mutation, MTO1 overexpression nearly fully restored τm^5^U frequency, partially increased aminoacylation efficiency, and upregulated mitochondrial protein synthesis and respiratory activity, although oxygen consumption showed only partial recovery due to continued degradation of structurally aberrant mt-tRNA^Leu(UUR)^ [144]. Similarly, overexpression of mitochondrial leucyl-tRNA synthetase (LARS2) or its C-terminal domain partially restored mitochondrial function in MELAS cells, likely through stabilization of pathogenic mt-tRNA molecules [13,145]. In MERRF patient cells carrying the A8344G mutation, overexpression of either MTO1 or TRMT61B—a tRNA methyltransferase catalyzing m^1^A at position 58—nearly completely restored mitochondrial translation, with TRMT61B demonstrating this effect without altering mt-tRNA^Lys^ [139]. For RILF-associated MTU1 mutations, where defective 2-thiolation results from CLPX-CLPP protease-mediated degradation, transient MTU1 enhancement or pharmacological CLPP inhibition may offer viable therapeutic approaches, given that patient survival requires maintenance of at least 10% residual 2-thiolation activity [13,146]. Substrate supplementation strategies have also yielded clinically significant benefits. High-dose oral taurine supplementation, approved as a MELAS treatment in Japan in 2019, significantly reduced stroke-like episodes in phase III trials without adverse effects, though progressive brain atrophy persisted, indicating symptomatic rather than curative benefit [147,148]. Similarly, L-cysteine or N-acetylcysteine supplementation shows potential in TRMU deficiency, while queuine supplementation may ameliorate abnormal tRNA cleavage in queuosine modification disorders [13].

Complementing modification-directed strategies, emerging genome editing technologies and RNA-based therapeutics offer unprecedented opportunities for precision correction of pathogenic variants at the DNA level. Mitochondrial-targeted base editors, including DdCBE (DddA-derived cytosine base editors) capable of C-to-T conversions and TALED systems (TALE-linked deaminases) enabling A-to-G conversions, have demonstrated proof-of-concept efficacy in cellular and animal models for directly correcting pathogenic mutations or shifting heteroplasmy toward wild-type mtDNA [149,150]. Mitochondrial-targeted nucleases (mitoTALENs, mitoZFNs) can selectively degrade mutant mtDNA molecules, promoting compensatory replication of wild-type genomes, while genetic suppression strategies—such as introduction of the T3290C compensatory mutation that restores τm^5^U modification in MELAS—illustrate the potential for mutation-specific correction approaches [149,151,152,153]. Additional strategies under investigation include suppressor tRNA approaches, allotopic expression of mitochondrial genes in the nucleus with mitochondrial targeting sequences, and pharmacological chaperones that stabilize mutant tRNAs. However, significant translational barriers persist, including challenges in mitochondrial delivery, off-target effects, immune responses, achieving sufficient heteroplasmy shift thresholds, and the scarcity of disease-relevant animal models for preclinical validation. Optimization of delivery vectors, enhancement of tissue specificity, comprehensive safety profiling, definition of therapeutic heteroplasmy thresholds, and establishment of regulatory frameworks remain critical priorities for advancing these promising therapeutic modalities from bench to bedside. As these technologies mature and our mechanistic understanding of mt-tRNA modification systems deepens, targeted correction of pathogenic mt-tRNA mutations holds transformative potential for the future of mitochondrial medicine [154,155,156,157].

Several future research priorities emerge from this comprehensive analysis. First, large-scale population-based sequencing initiatives are essential to define the true prevalence of pathogenic mt-tRNA variants and clarify penetrance patterns across diverse ethnic backgrounds [24]. Second, improved functional assays derived from induced pluripotent stem cells and CRISPR-edited cellular models are needed to distinguish truly pathogenic mutations from benign polymorphisms, particularly for variants of uncertain significance [158]. Finally, the development of robust genotype-phenotype prediction algorithms incorporating heteroplasmy levels, nuclear genetic modifiers, and clinical biomarkers will enhance personalized prognostic counseling and therapeutic decision-making [91].

In conclusion, this systematic review provides an updated and comprehensive classification of pathogenic mt-tRNA mutations and their associated disease phenotypes. By consolidating genetic, clinical, and mechanistic data across the full spectrum of mt-tRNA-related disorders, this analysis can serve as a useful reference framework for clinicians, genetic counselors, and researchers. As next-generation sequencing has now become affordably accessible and genome editing technologies mature, the ability to accurately identify, interpret, and ultimately correct pathogenic mt-tRNA mutations will transform the diagnostic and therapeutic landscape for millions of individuals worldwide affected by mitochondrial dysfunction caused by mt-tRNA mutations. The comprehensive overview presented herein can serve not only as a critical resource for clinical practice but also as a guide for future precision interventions. Key priorities for future research include functional validation of uncharacterized mt-tRNA variants and development of standardized pathogenicity criteria to support accurate diagnosis. Large-scale clinical studies are also needed to clarify genotype–phenotype relationships. Completing the map of mitochondrial tRNA modification pathways remains a major unmet need, as several enzymes are still unidentified. Finally, advancing and carefully assessing mtDNA-editing tools will be critical for translating these mechanistic insights into targeted therapies.

## Data Availability

No new data were created or analyzed in this study. Data sharing is not applicable to this article.

## Abbreviations

The following abbreviations are used in this manuscript:

ATP	Adenosine triphosphate
AD	Alzheimer’s disease
Ago2	Argonaute 2
AC	Arrhythmogenic cardiomyopathy
ASD	Autism Spectrum Disorders
APS2	Autoimmune polyendocrinopathy type II
CKD	Chronic kidney disease
CLPX-CLPP	Caseinolytic protease
CPEO	Chronic progressive external ophthalmoplegia
CTD	Chronic tic disorder
CoQ10	Coenzyme Q10
CAD	Coronary artery disease
Cyt b	Cytochrome b
COX1	Cytochrome c oxidase 1
COX2	Cytochrome c oxidase 2
COX3	Cytochrome c oxidase 3
DdCBE	DddA-derived cytosine base editors
DCM	Dilated cardiomyopathy
EH	Essential hypertension
FSGS	Focal segmental glomerulosclerosis
HCM	Hypertrophic cardiomyopathy
KSS	Kearns–Sayre syndrome
LHON	Leber’s hereditary optic neuropathy
LARS2	Leucyl-tRNA synthetase 2
LVNC	Left ventricular noncompaction cardiomyopathy
MDD	Major depressive disorder
MIDD	Maternally inherited diabetes and deafness
mtDNA	Mitochondrial DNA
MDs	Mitochondrial diseases
MELAS	Mitochondrial Encephalomyopathy, Lactic Acidosis, and Stroke-like episodes
MMP	Mitochondrial membrane potential
MM	Mitochondrial myopathies
MNGIE	Mitochondrial Neurogastrointestinal Encephalopathy
mtSNPs	Mitochondrial single nucleotide polymorphisms
mitoTALENS	Mitochondrial transcription activator-like effector nucleases
MITKD	Mitochondrial Tubulointerstitial Kidney Disease
mt-tRNAs	Mitochondrial tRNAs
MT-TA	Mitochondrial tRNAAla gene
MT-TR	Mitochondrial tRNAArg gene
MT-TN	Mitochondrial tRNAAsn gene
MT-TD	Mitochondrial tRNAAsp gene
MT-TC	Mitochondrial tRNACys gene
MT-TQ	Mitochondrial tRNAGln gene
MT-TE	Mitochondrial tRNAGlu gene
MT-TG	Mitochondrial tRNAGly gene
MT-TH	Mitochondrial tRNAHis gene
MT-TI	Mitochondrial tRNAIle gene
MT-TL2	Mitochondrial tRNALeu(CUN) gene
MT-TL1	Mitochondrial tRNALeu gene
MT-TK	Mitochondrial tRNALys gene
MT-TM	Mitochondrial tRNAMet gene
MT-TF	Mitochondrial tRNAPhe gene
MT-TP	Mitochondrial tRNAPro gene
MT-TS2	Mitochondrial tRNASer(AGY) gene
MT-TS1	Mitochondrial tRNASer(UCN) gene
MT-TT	Mitochondrial tRNAThr gene
MT-TW	Mitochondrial tRNATrp gene
MT-TY	Mitochondrial tRNATyr gene
MT-TV	Mitochondrial tRNAVal gene
MDS	Myelodysplastic Syndrome
MERRF	Myoclonic Epilepsy with Ragged Red Fibers
ND1	NADH dehydrogenase 1
ND2	NADH dehydrogenase 2
ND3	NADH dehydrogenase 3
ND4	NADH dehydrogenase 4
ND4L	NADH dehydrogenase 4L
ND5	NADH dehydrogenase 5
ND6	NADH dehydrogenase 6
NSHL	Non-Syndromic Sensorineural Hearing Loss
OXPHOS	Oxidative phosphorylation
PD	Parkinson’s disease
PCOS	Polycystic Ovary Syndrome
PTD	Provisional tic disorder
ROS	Reactive oxygen species
RILF	Reversible infantile liver failure
rRNAs	Ribosomal RNAs
RISC	RNA-induced silencing complex
SLE	Systemic Lupus Erythematosus
TALEDs	TALE-linked deaminases
DSM-V	The Diagnostic and Statistical Manual of Mental Disorders, Fifth Edition
TD	Tic Disorders
tRNAs	Transfer RNAs
T2DM	Type 2 Diabetes Mellitus
